# Stress-Activated Degradation of Sphingolipids Regulates Mitochondrial Function and Cell Death in Yeast

**DOI:** 10.1155/2017/2708345

**Published:** 2017-08-06

**Authors:** Sara Manzanares-Estreder, Amparo Pascual-Ahuir, Markus Proft

**Affiliations:** ^1^Department of Molecular and Cellular Pathology and Therapy, Instituto de Biomedicina de Valencia (IBV-CSIC), Jaime Roig 11, 46010 Valencia, Spain; ^2^Department of Biotechnology, Instituto de Biología Molecular y Celular de Plantas, Universitat Politècnica de València-CSIC, Ingeniero Fausto Elio s/n, 46022 Valencia, Spain

## Abstract

Sphingolipids are regulators of mitochondria-mediated cell death in higher eukaryotes. Here, we investigate how changes in sphingolipid metabolism and downstream intermediates of sphingosine impinge on mitochondrial function. We found in yeast that within the sphingolipid degradation pathway, the production via Dpl1p and degradation via Hfd1p of hexadecenal are critical for mitochondrial function and cell death. Genetic interventions, which favor hexadecenal accumulation, diminish oxygen consumption rates and increase reactive oxygen species production and mitochondrial fragmentation and vice versa. The location of the hexadecenal-degrading enzyme Hfd1p in punctuate structures all along the mitochondrial network depends on a functional ERMES (endoplasmic reticulum-mitochondria encounter structure) complex, indicating that modulation of hexadecenal levels at specific ER-mitochondria contact sites might be an important trigger of cell death. This is further supported by the finding that externally added hexadecenal or the absence of Hfd1p enhances cell death caused by ectopic expression of the human Bax protein. Finally, the induction of the sphingolipid degradation pathway upon stress is controlled by the Hog1p MAP kinase. Therefore, the stress-regulated modulation of sphingolipid degradation might be a conserved way to induce cell death in eukaryotic organisms.

## 1. Introduction

Sphingolipids are specialized bioactive lipid molecules found in the membranes of all eukaryotic cells. They have critical functions in the control of cell growth, senescence, differentiation, and programmed cell death. Different intermediates of sphingolipid pathways can have opposing effects on cell signaling. Therefore, imbalances in sphingolipid metabolism can deregulate key cellular processes and contribute to human disorders [1–3]. A critical cellular homeostasis pathway regulated by sphingolipids is mitochondria-mediated apoptosis [4]. In this process, multiple pathways converge on mitochondria and induce mitochondrial outer membrane permeabilization (MOMP). MOMP causes the release of proteins from the mitochondrial intermembrane space into the cytosol, where they execute the programmed cell death via the caspase cascade in higher eukaryotes. Therefore, MOMP is considered the initial irreversible trigger of mitochondrial apoptosis [5]. This death decision needs to be tightly controlled and depends on the interplay of several pro- and antiapoptotic members of the Bcl-2 family [6, 7]. Some Bcl-2 protein members such as Bax have to gain access to mitochondria in response to apoptotic stimulation, as they are normally cytosolic proteins [8, 9]. During apoptotic activation, Bax structural conformation is altered in a regulated manner, which facilitates its insertion into the outer mitochondrial membrane followed by dimerization and the formation of higher order oligomeric pores to induce MOMP [10, 11]. Specific sphingolipid levels at mitochondria are known to regulate the entry into mitochondrial apoptosis [4, 12]. Ceramide has been identified as a prodeath molecule, as it can induce apoptosis when applied externally [13], and the regulation of ceramide biosynthesis and degradation is important for the entry into the intrinsic apoptotic pathway [14–16]. However, recent studies point to important proapoptotic functions for metabolites of the sphingolipid degradation pathway, such as sphingosine-1-phosphate (S1-P) or hexadecenal [17, 18]. S1-P and hexadecenal are efficient inducers of Bax-mediated apoptosis and MOMP in vitro [17]. Since sphingolipid metabolism takes place in the ER, the regulated production and transfer of sphingolipid metabolites to mitochondria might be an important, but yet unexplored, upstream event in the initiation of cell death [19].

Despite their simplicity, yeast cells are an informative model to understand the regulation of programmed cell death [20, 21]. Importantly, yeast undergoes MOMP, cytochrome *c* release, and mitochondrial fragmentation in response to apoptotic stimuli [22–24], as well as upon the heterologous expression of the human apoptosis executer Bax [25, 26]. As for higher eukaryotes, also in yeast, our information about the effect of sphingolipid metabolism on mitochondrial function and death is limited to ceramide levels [27, 28]. The lack of enzyme functions leading to ceramide production has been shown to decrease programmed cell death in response to acetic acid in yeast [29]. The enzymes involved are Isc1p and Lag1p, which contribute to ceramide formation by the breakdown of complex sphingolipids or by de novo synthesis, respectively. However, the recent identification of the precise enzymatic steps involved in the degradation of sphingolipids and the formation of the key intermediates S1-P and hexadecenal in yeast [30] has enabled us to study the effect of this metabolic pathway on mitochondrial-mediated cell death. We show here that the key enzyme in this pathway, the hexadecenal metabolizing Hfd1p dehydrogenase, has a stress-regulated function associated with mitochondria, which might be decisive for the entry into mitochondrial cell death.

## 2. Materials and Methods

### 2.1. Yeast Strains and Plasmids

A complete list of yeast strains used in this work can be found in Table 1. Constitutive expression of Hfd1-GFP was obtained by cloning full length *HFD1* into the yeast Gateway vector pAG426-GPD-ccdB-GFP [31]. Fusion of full length *OM14* with dsRed was performed in yeast Gateway vector pAG415-GPD-ccdB-dsRed [31]. Galactose inducible overexpression of Dpl1p was achieved by cloning full length *DPL1* into yeast Gateway vector pAG426-GAL1p-ccdB-HA [31]. Plasmids pCM189-Bax and pCM184-Bcl-x_L_ for Tet_off_-controlled Bcl-2 member protein expression were a kind gift from S. Manon [32]. GFP-Ybh3 fusion protein expression was achieved from plasmid pUG36-GFP-YBH3 (a kind gift from F. Madeo [33]). For the galactose inducible expression of a Mmm1-mCherry fusion protein, plasmid pAG413-GAL1p-MMM1-mCherry was used (a kind gift from W. Prinz [34]).

### 2.2. Growth Conditions

Yeast strains were grown at 28°C in yeast extract peptone containing 2% dextrose (YPD) or 2% galactose (YPGal) for agar plate assays. Synthetic dextrose (SD) or galactose (SGal) media contained 0.67% yeast nitrogen base, 50 mM succinic acid (pH 5.5), and 2% of the respective energy source. According to the auxotrophies of each strain, methionine (10 mg/l), histidine (10 mg/l), leucine (10 mg/l), or uracil (25 mg/l) were added. As indicated, growth media were supplemented with menadione, valinomycin, or NaCl from appropriate stocks in DMSO (menadione) or water (valinomycin, NaCl). Yeast strains transformed with Bax- or Bcl-x_L_-expression plasmids were pregrown in the presence of 10 *μ*g/ml doxycycline (Sigma), and protein expression was induced by switching cells to doxycycline-free medium. For sensitivity assays with externally added sphingolipid metabolic intermediates, serial dilutions of the cells were treated in synthetic liquid medium with the indicated doses of the compounds from DMSO stock solutions and then plated onto YPD agar plates. Palmitic acid, hexadecenoic acid, and sphingosine-1-phosphate were purchased from Sigma; hexadecenal was purchased from Avanti Polar Lipids. For quantitative colony assays, fresh overnight yeast cultures were diluted in triplicate to OD_600_ = 0.1 and allowed to grow for 24 h on the indicated media. Alternatively, for the hexadecenal toxicity assays, fresh overnight cultures in SD medium were adjusted to the same OD_600_ and then treated with the indicated concentrations of hexadecenal for 2 h. The number of colony-forming units was then determined by plating the cultures onto YPD agar plates at appropriate dilutions.

### 2.3. Reverse Transcriptase Assays

Total RNA was isolated by acid phenol extraction from yeast cells grown in the indicated conditions. RNA samples were digested with DNaseI and purified with the RNeasy Mini kit (Qiagen). A total of 5 *μ*g of RNA was converted into DNA using the SuperScript III First-Strand cDNA Synthesis kit (Invitrogen). The amount of DNA was determined with the indicated gene-specific primers by quantitative PCR in real time using the EvaGreen qPCR Master Mix (Biotium) on an Applied Biosystems 7500 sequence detection system. The *ACT1* gene was used as a reference. Relative expression levels were calculated in triplicate from three independent cDNA samples. Primer sequences are available upon request.

### 2.4. Microscopy

Mitochondria were visualized with MitoTracker Red CMXRos (Invitrogen). Cells were incubated with 1 *μ*M MitoTracker dye for 60 min, washed once, and finally resuspended in fresh culture medium. Cells were observed on a Leica confocal microscope TCS SP8 using the following excitation and emission wavelengths: GFP (excitation 488 nm; emission 509 nm), mCherry (excitation 587 nm; emission 610 nm), dsRed (excitation 560 nm; emission 592 nm), and MitoTracker Red (excitation 578 nm; emission 600 nm).

### 2.5. ROS Determination

Exponential cell culture aliquots upon the indicated growth conditions were incubated for 30 min with 2′,7′-dichlorodihydrofluorescein diacetate (Sigma) at a final concentration of 10 *μ*M. The cells were washed with water and resuspended in 1 ml of 50 mM Tris/HCl pH 7.5. After the addition of 10 *μ*l of chloroform and 5 *μ*l of 0.1% SDS, cell extracts were prepared by rigorous agitation with glass beads. Fluorescence was quantified in the supernatant in a microplate reader at 492 nm excitation and 525 nm emission wavelengths and normalized for the fluorescence of the same number of mock-treated cells.

### 2.6. Measurement of Oxygen Consumption

For measurements of respiration rates, yeast cells were grown to exponential phase in synthetic galactose-containing medium, washed with water, and finally resuspended at the same OD in 40 mM NaPO_4_ pH 7.4 with 1% glucose. Oxygen consumption was then quantified in intact cells using a Mitocell S200 Respirometry System (Strathkelvin Instruments) with a Clarke type oxygen electrode. Oxygen consumption rates were determined from at least three independent yeast cultures for each strain background.

### 2.7. Statistical Analysis

Student's *t*-test was performed to compare the mean values between two different conditions or strains. A *P* value < 0.05 was considered statistically significant.

## 3. Results and Discussion

### 3.1. The Sphingolipid Degradation Pathway Regulates Mitochondrial Function

**Figure 1:**
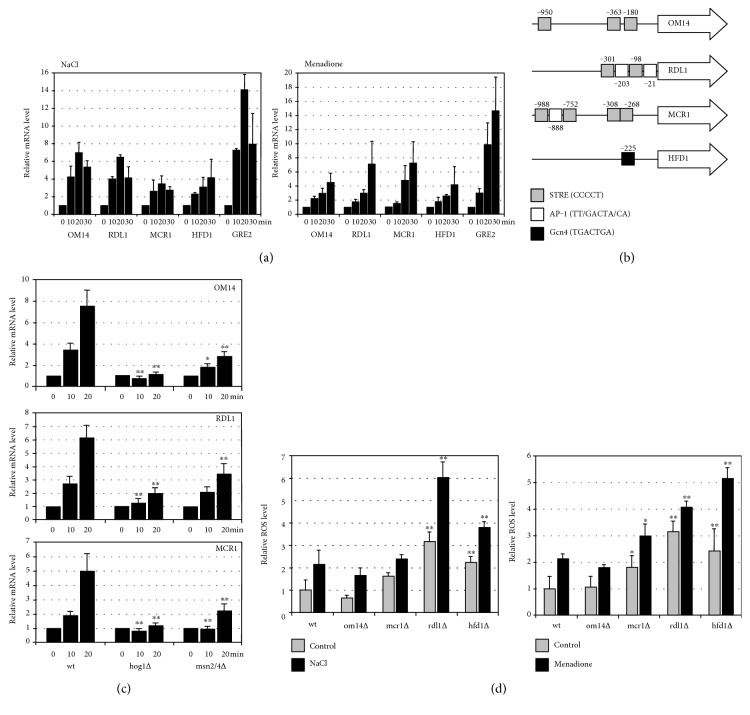
Stress-induced expression of genes encoding outer mitochondrial membrane proteins. (a) Reverse transcriptase determination of mRNA induction of four outer mitochondrial membrane protein encoding genes upon salt shock (0.4 M NaCl) and oxidative stress (50 *μ*M menadione) for the indicated times. The *GRE2* gene was included as a positive marker for salt and oxidative stress. Data are presented as mean ± SD. Three biological replicates were analyzed. The mRNA level was normalized in all cases for the *ACT1* control, and the uninduced level was arbitrarily set to 1. (b) Representation of consensus binding sites for stress-activated transcription factors in the promoter regions of *OM14*, *RDL1*, *MCR1*, and *HFD1*. (c) Reverse transcriptase determination of mRNA induction of the same genes in the indicated strain backgrounds upon salt shock (0.4 M NaCl) as described in (a). Significantly different mRNA levels as compared to wt are marked. ^∗^*P* < 0.05; ^∗∗^*P* < 0.01 (Student's *t*-test). (d) Reactive oxygen species (ROS) production in mutants affected in specific mitochondrial outer membrane proteins. 2′,7′-dichlorodihydrofluorescein diacetate assay in the indicated yeast strains before or after salt (1 M NaCl, 2 h) or oxidative shock (50 *μ*M menadione, 2 h). Data are presented as mean ± SD. Three biological replicates were analyzed. Significantly different ROS levels as compared to wt are marked. ^∗^*P* < 0.05; ^∗∗^*P* < 0.01 (Student's *t*-test).

It has been previously shown that salt stress causes a profound change in mitochondrial activity [35, 36]. Certain outer mitochondrial membrane proteins such as Hfd1p, Om14p, Rdl1p, or Mcr1p seem to specifically accumulate under these conditions at the organelle [35]. Om14p is a mitochondrial receptor for ribosomes with a function in the cotranslational import of mitochondrial proteins [37], Rdl1p is a putative mitochondrial thiosulfate sulfurtransferase [38], while Mcr1p is a mitochondrial NADH-cytochrome b5 reductase with a known function in cellular ROS homeostasis [39]. We tested whether the expression of the corresponding nuclear genes was regulated upon stress. All four genes were several fold upregulated upon both salt and oxidative stress (Figure 1(a)). Further inspection of the promoter regions of the four genes revealed that only *OM14*, *RDL1*, and *MCR1* contained several classical stress response elements (STRE; Figure 1(b)). Indeed, the stress-dependent transcriptional control of the three genes was lost in the absence of the STRE-binding activators Msn2p/Msn4p or the stress-activated MAP kinase Hog1p (Figure 1(c)). An important function of the modulation of mitochondrial activity during salt stress is the maintenance of ROS homeostasis [36]. The ROS production was quantitatively assessed in the respective yeast mutant strains upon normal growth and salt or oxidative challenge. We found that the absence of Hfd1p or Rdl1p caused a major ROS imbalance (Figure 1(d)). We further investigated the role of Hfd1p in the stress-regulated control of mitochondrial function.

**Figure 2:**
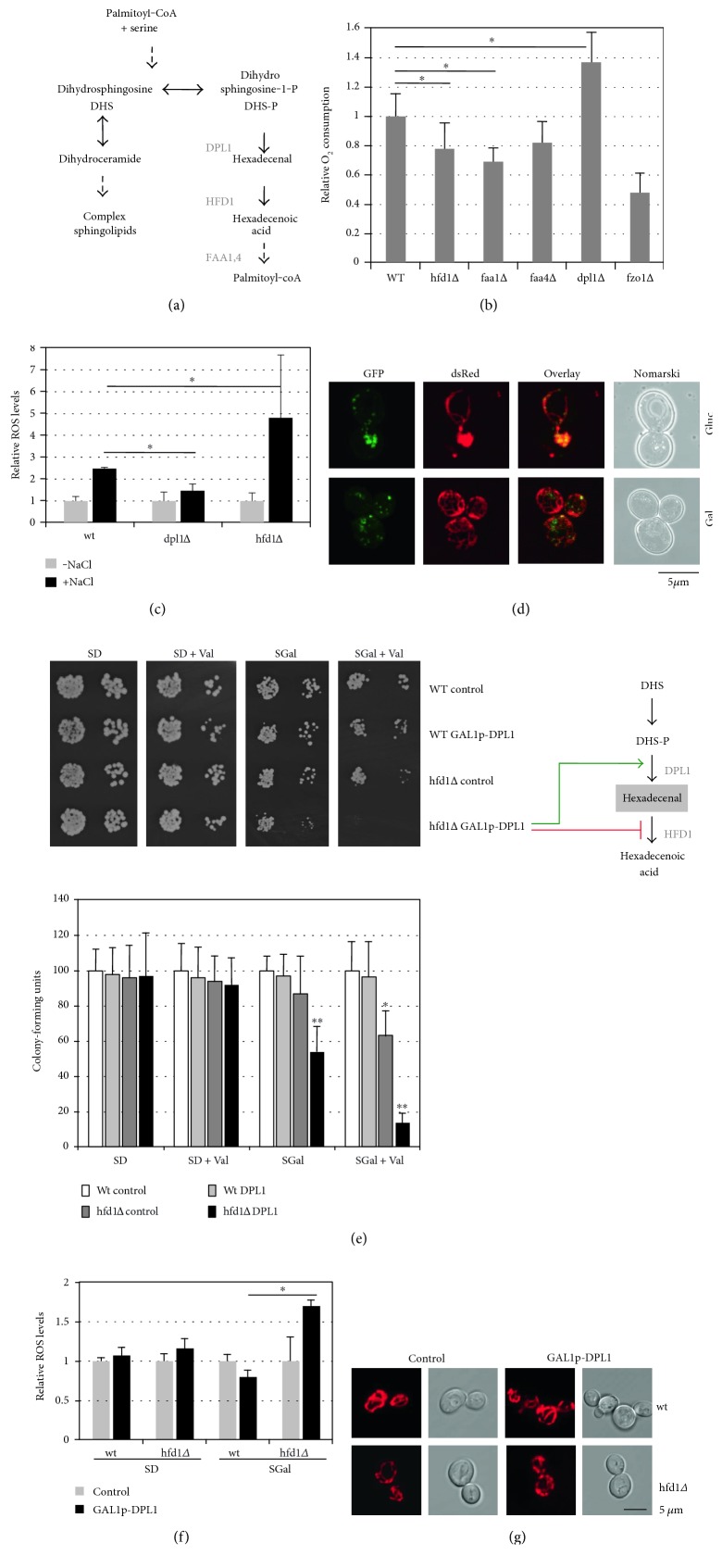
Sphingolipid degradation modulates mitochondrial activity in yeast. (a) Schematic overview of the enzymatic conversions of sphingolipid degradation. Downstream enzymatic activities of dihydrosphingosine-1-phosphate are depicted. Only the conversions of dihydrosphingosine and not of other sphingosine species such as phytosphingosine are shown. (b) Oxygen consumption rates of mutants affected in the sphingolipid degradation pathway. Cells were grown in synthetic galactose medium. The *fzo1Δ* mutant was included as a negative control. The O_2_ consumption rate of the wild type was arbitrarily set to 1. (c) Reactive oxygen species (ROS) production in mutants affected in hexadecenal production (*dpl1Δ*) or degradation (*hfd1Δ*). 2′,7′-dichlorodihydrofluorescein diacetate assay in the indicated yeast strains before or after salt shock (1 M NaCl, 2 h). ROS levels upon normal growth conditions were set to 1 for each strain background. (d) Intracellular localization of Hfd1p. Cells expressing constitutive Hfd1-GFP and Om14-dsRed fusion proteins were grown in synthetic glucose- or galactose-containing medium. (e, f) Genetic manipulation of the sphingolipid degradation pathway affects cell viability and ROS production. The hexadecenal-producing Dpl1p enzyme was overexpressed under control of the *GAL1* promoter in yeast wild type or the *hfd1Δ* mutant. (e) Growth efficiency was assessed on synthetic agar medium-containing glucose (SD) or galactose (SGal) supplemented or not with 4 *μ*M valinomycin. Alternatively, colony formation was quantified in the same strains (lower panel). Cells from fresh overnight cultures in synthetic glucose medium were diluted in the indicated media to an OD_600_ of 0.1, and growth was allowed for an additional 24 h. Colony-forming units were determined by plating the cells onto YPD agar medium. The colony number obtained for the wt upon the different growth conditions was set to 100. (f) Quantification of ROS production in the same strains grown in synthetic glucose or galactose medium by the 2′,7′-dichlorodihydrofluorescein diacetate assay. (g) Overexpression of Dpl1p causes mitochondrial fragmentation in *hfd1Δ* mutants. MitoTracker-stained mitochondria were visualized in the indicated yeast cells containing the empty vector or the galactose-inducible *DPL1* expression on synthetic galactose medium. Data information: in (b, c, e, and f), data are presented as mean ± SD. Three biological replicates were analyzed. Significant changes with respect to the wild type are marked. ^∗^*P* < 0.05, ^∗∗^*P* < 0.01 (Student's *t*-test).

*HFD1* encodes a fatty aldehyde dehydrogenase, which catalyzes the conversion of hexadecenal to hexadecenoic acid in the sphingosine-1-phosphate (S1-P) degradation pathway [30]. Sphingolipids are well-known inducers of programmed cell death in higher eukaryotes and mitochondrial dysfunction in yeast [4, 27]. We wanted to determine whether downstream metabolites resulting from sphingolipid degradation were able to modulate mitochondrial function in yeast (Figure 2(a)). We found that genetic interruption of the pathway upstream of hexadecenal formation in the *dpl1Δ* mutant increased oxygen consumption rates of whole yeast cells. A block after hexadecenal formation in the *hfd1Δ* or *faa1Δ* mutants decreased oxygen consumption (Figure 2(b)). This result was further confirmed by a decreased ROS production in the *dpl1Δ* mutant upon stress as opposed to the previously shown overproduction of ROS in the *hfd1Δ* strain (Figure 2(c)). These data suggested that the hexadecenal-metabolizing enzyme Hfd1p had a function in modulating mitochondrial activity and cellular ROS levels. We next determined the subcellular localization of Hfd1p and found that the enzyme localized to multiple spots along the whole mitochondrial network (Figure 2(d)). These punctuate structures containing Hfd1p were equally observed after activation of mitochondrial biogenesis upon growth on galactose medium.

If intracellular hexadecenal levels triggered mitochondrial dysfunction and oxidative stress, then genetic manipulations of the sphingosine 1-phosphate metabolic pathway, which favor the accumulation of hexadecenal, should affect cell viability. To test this hypothesis, we conditionally overexpressed the hexadecenal-generating enzyme Dpl1p in wild-type and *hfd1Δ* cells. While the Dpl1p gain of function did not lead to any growth phenotype in a wild type, it inhibited growth in the *hfd1Δ* mutant (Figure 2(e)) accompanied by ROS overproduction (Figure 2(f)), and this inhibition was further increased upon mitochondrial damage caused by valinomycin. Additionally, mitochondria are slightly more fragmented in a *hfd1Δ* background alone, while mitochondrial fragmentation is further increased after Dpl1p overexpression (Figure 2(g)). Thus, modulation of sphingolipid metabolism, and in particular the hexadecenal intermediate, seemed to regulate mitochondrial function and cell growth.

### 3.2. Hexadecenal Is the Main Inducer of Mitochondrial Dysfunction and Fragmentation

**Figure 3:**
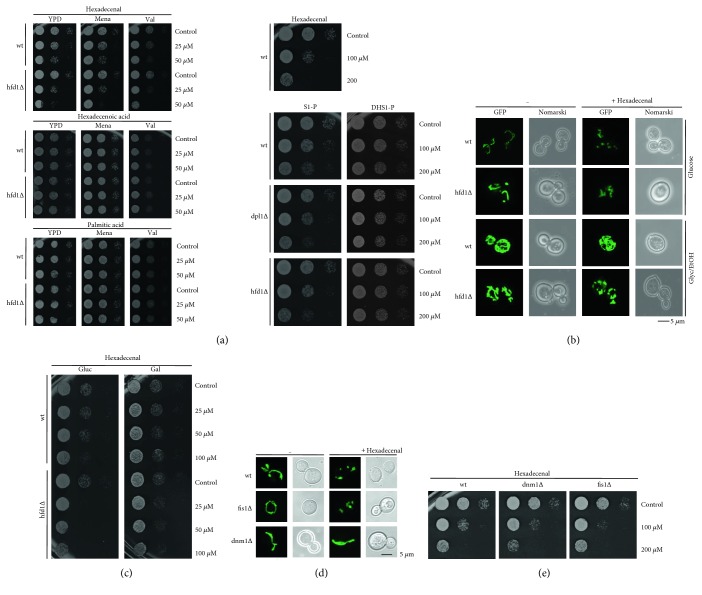
Hexadecenal is the most biologically active intermediate of the sphingolipid degradation pathway. (a) Hexadecenal and the downstream metabolites hexadecenoic and palmitic acid were tested for growth inhibition of the indicated yeast strains (left panel). The indicated doses were applied for 2 h, and colony formation was subsequently assessed on YPD plates containing or not 50 *μ*M menadione or 4 *μ*M valinomycin. The upstream metabolites sphingosine-1-phosphate (S1-P) and dihydrosphingosine-1-phosphate were tested for growth inhibition of the indicated yeast strains in the right panel. (b) External hexadecenal addition causes mitochondrial fragmentation dependent on Hfd1p function. Mitochondria were visualized by expression of mt-GFP in yeast wild type and the *hfd1Δ* mutant in synthetic glucose or glycerol/ethanol medium before and after the exposure (1 h) to 50 *μ*M hexadecenal. (c) Galactose growth counteracts hexadecenal growth inhibition. Hexadecenal was applied for 1 h to the indicated yeast strains grown on glucose- or galactose-containing synthetic medium. Colony formation was then assessed on YPD agar plates. (d) Hexadecenal induces mitochondrial fragmentation through Dnm1p. The indicated yeast strains expressing mt-GFP were treated or not with 50 *μ*M hexadecenal for 1 h before visualization of mitochondria. (e) Suppression of mitochondrial fission does not counteract hexadecenal-mediated growth inhibition. The indicated yeast strains were assayed for hexadecenal inhibition as in (a).

The effect of hexadecenal on cell growth was tested directly by external addition to yeast cells. As shown in Figure 3(a), hexadecenal strongly inhibited cell growth in wild-type cells, which was slightly exacerbated by oxidative stress or mitochondrial damage. Hfd1p is important to counteract hexadecenal toxicity, which is demonstrated by the hypersensitivity of *hfd1Δ* cells to hexadecenal (Figure 3(a)). Downstream metabolites such as hexadecenoic or palmitic acid did not cause growth inhibition demonstrating the specificity of hexadecenal. The upstream metabolites sphingosine-1-P or dihydrosphingosine-1-P inhibited growth in a significantly weaker manner independently of the downstream enzymatic activities of Dpl1p and Hfd1p (Figure 3(a)). Thus, within the S1-P degradation pathway, hexadecenal is the most biologically active metabolite. Accordingly, hexadecenal treatment rapidly induced mitochondrial fragmentation preferentially in glucose-grown cells. A fully developed mitochondrial network upon respiratory growth seemed to protect from hexadecenal-induced fragmentation in wild-type cells, but not in the *hfd1* mutant (Figure 3(b)). Additionally, galactose growth counteracted hexadecenal-induced growth inhibition, which was still observed in *hfd1Δ* mutants (Figure 3(c)).

We further found that the hexadecenal-induced mitochondrial fragmentation depended on the Dnm1p GTPase and not the Fis1p fission protein (Figure 3(d)). However, mitochondrial fission is not the reason for hexadecenal-triggered cell death, because the *dnm1Δ* strain was even more sensitive to the fatty aldehyde than wild-type cells (Figure 3(e)). Taken together, hexadecenal efficiently triggers cell death in yeast cells, which is not causally linked to mitochondrial fragmentation. Accordingly, although mitochondrial fragmentation generally accompanies apoptosis in different cellular models, it is not a prerequisite for cell death [40]. In mammalian cells, the Drp1 fission protein is responsible for apoptotic mitochondrial fragmentation, but similarly to what is reported here for yeast, its function in mitochondrial fission is not decisive for MOMP and subsequent cell death [41, 42].

### 3.3. Modulation of Sphingolipid Metabolism Triggers Mitochondrial Cell Death in Yeast

**Figure 4:**
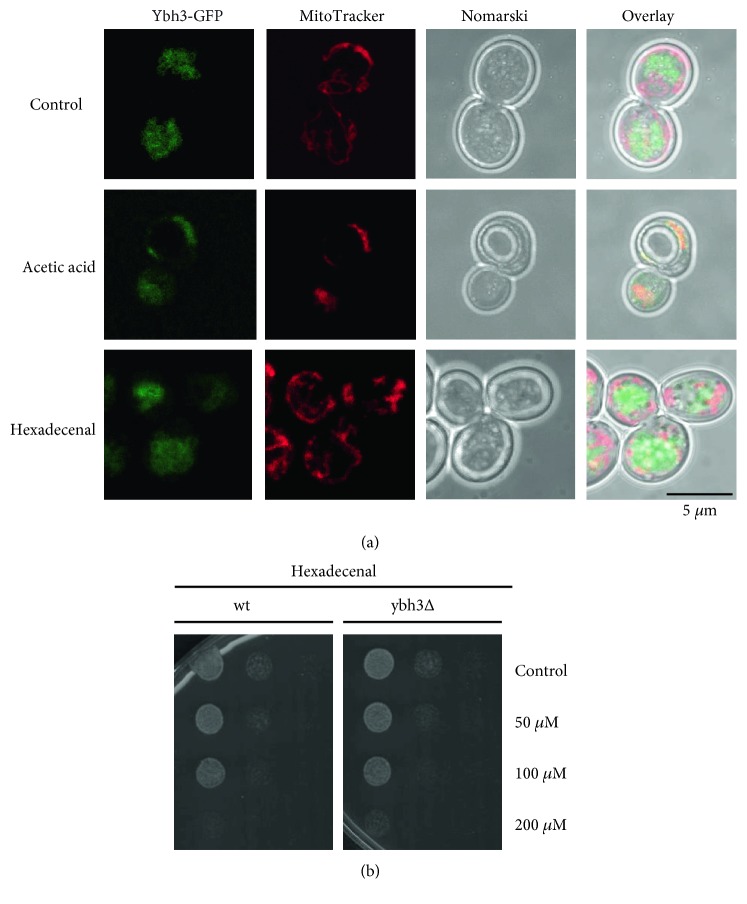
Yeast Ybh3p is not involved in hexadecenal-mediated cell death. (a) Mitochondrial association of Ybh3p is stimulated by acetic acid, but not by hexadecenal. Intracellular localization relative to the MitoTracker-stained mitochondria of a Ybh3-GFP fusion protein expressed in wild type was observed upon acetic acid (100 mM, 1 h at pH 3.0) or hexadecenal (50 *μ*M, 1 h) treatment. (b) Sensitivity to hexadecenal is not altered in *ybh3Δ* mutants. The growth efficiency upon external addition of hexadecenal was assayed as in Figure 3(a) in wild type and *ybh3Δ* mutants.

We next wanted to test whether the sphingolipid degradation pathway and especially the hexadecenal intermediate triggered cell death via the mitochondrial death pathway. In higher eukaryotes, mitochondrial apoptosis is irreversibly induced by the permeabilization of the outer mitochondrial membrane. This process is highly controlled by pro- and antiapoptotic members of the Bcl-2 family. In yeast, only one Bcl-2 ortholog has been identified, Ybh3p, which has both pro- and antiapoptotic properties [33, 43]. We tested whether Ybh3p was functionally involved in hexadecenal-mediated growth arrest. As expected, Ybh3p translocated to mitochondria upon the apoptotic stimulus of acetic acid treatment. However, hexadecenal treatment did not induce mitochondrial localization of Ybh3p (Figure 4(a)). Additionally, the lack of Ybh3p function did not change the susceptibility of yeast cells to externally added hexadecenal (Figure 4(b)). Ybh3p is therefore not the executor of hexadecenal-induced cell death.

**Figure 5:**
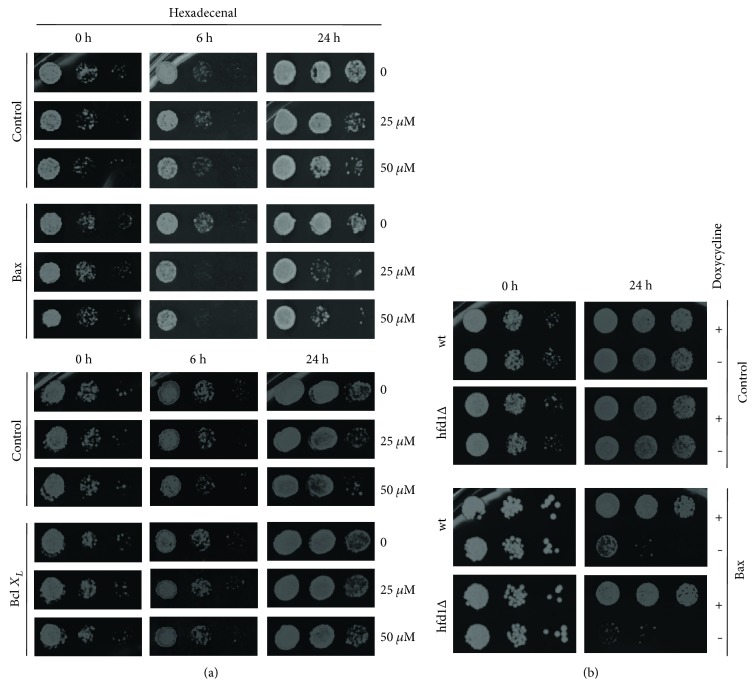
Bax-mediated growth inhibition is modulated by hexadecenal levels. (a) External hexadecenal potentiates Bax function. Human proapoptotic Bax and antiapoptotic Bcl-x_L_ were expressed under control of the Tet_off_ promoter for the indicated times in the presence or not of the indicated hexadecenal concentrations. Control strains contained the respective empty vectors. Colony formation was then assessed on YPD agar plates. (b) Bax inhibition is enhanced in *hfd1Δ* mutants. Human Bax expression was induced for 24 h in the indicated yeast strains by the removal of doxycycline.

Alternatively, we examined the death-promoting and death-preventing functions of human Bcl-2 members in yeast cells with an altered sphingolipid metabolism. Human Bax protein has been shown to efficiently activate mitochondrial cell death in yeast [25, 44]. Here, we found that the induced Bax expression arrested cell growth in a synergistic manner with external hexadecenal (Figure 5(a)). On the other hand, antiapoptotic human Bcl-x_L_ expression largely prevented hexadecenal-induced growth inhibition. Thus, hexadecenal seemed to arrest cell growth via proteins of the Bcl-2 family when expressed ectopically in yeast. This was further supported by the fact that Bax was a more efficient inhibitor in cells with a block in the hexadecenal-metabolizing Hfd1p enzyme (Figure 5(b)). Thus, Bcl-2 proteins such as Bax promote growth arrest in yeast cells in a process, which is modulated by sphingolipid degradation products such as hexadecenal. Our results support the idea that high mitochondrial hexadecenal levels would favor the proapoptotic action of Bax and subsequently promote mitochondrial fragmentation. In this model, the Hfd1p enzyme would counteract hexadecenal accumulation at mitochondria and prevent the induction of cell death. It remains to be experimentally addressed whether changes in hexadecenal metabolism trigger mitochondrial outer membrane permeabilization in yeast.

**Figure 6:**
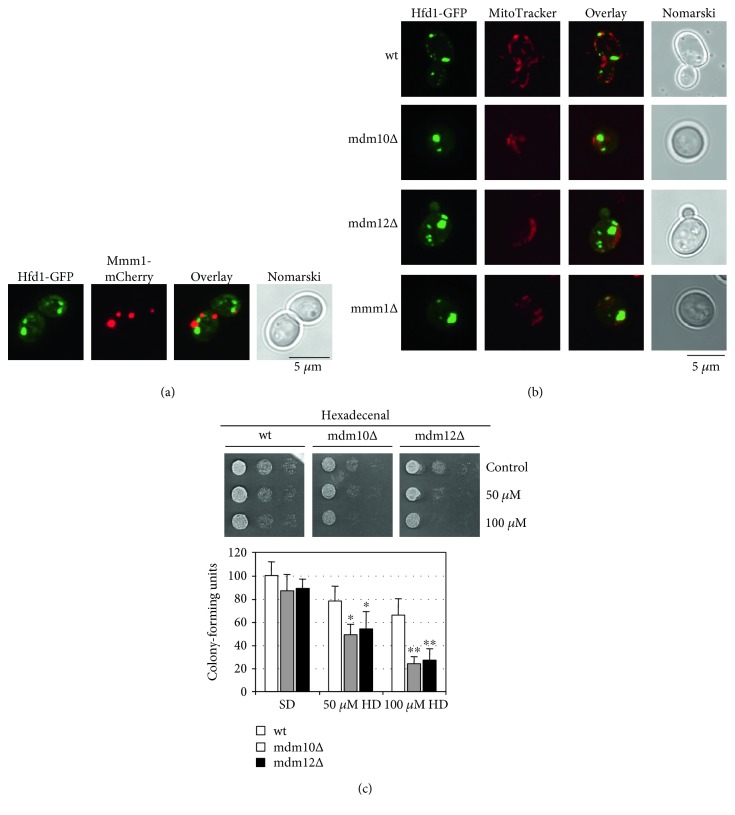
Functional connection between the ERMES complex and hexadecenal-mediated cell death. (a) Colocalization study of Hfd1-GFP with the Mmm1-mCherry ERMES complex subunit. Cells were grown in synthetic galactose medium for the induced expression of the Mmm1-mCherry fusion. (b) Intracellular distribution of Hfd1p is affected in ERMES complex mutants. Hfd1-GFP was expressed in the indicated yeast strains and localized relative to MitoTracker-stained mitochondria. Cells were grown in synthetic glucose medium. (c) ERMES complex mutants are hypersensitive to hexadecenal. The growth efficiency upon external addition of hexadecenal (HD) was assayed as in Figure 3(a) in wild type and the indicated ERMES deletion mutants (upper panel). Quantitative colony assays are shown for the same strains in the lower panel. Cells from fresh overnight cultures in synthetic glucose medium were diluted to OD_600_ 0.5 and then incubated with the indicated hexadecenal doses for 2 h. Colony-forming units were determined by plating the cells onto YPD agar medium. Data are presented as mean ± SD. Three biological replicates were analyzed. The colony number obtained for the wt upon control conditions was set to 100. Significant changes with respect to the wild type upon the same growth condition are marked. ^∗^*P* < 0.05, ^∗∗^*P* < 0.01 (Student's *t*-test).

As Hfd1p localizes to specific sites along mitochondria and the metabolism of complex sphingolipids occurs at the endoplasmic reticulum, we tested whether ER-mitochondrial contacts were involved in the hexadecenal-mediated cell death. The ER-mitochondria encounter structure (ERMES), which tethers mitochondria to the ER at strategic sites [45], has been implied in the transfer of phospholipids from the ER to mitochondria [46]. However, a direct role of ERMES in lipid transfer between the two organelles is still a matter of debate [47]. We found that Hfd1p often localizes in close vicinity to the ERMES subunit Mmm1p (Figure 6(a)). Moreover, the distribution of Hfd1p in small spots along mitochondria is disrupted in several ERMES mutants, and an aberrant distribution of Hfd1p in very few enlarged structures was observed (Figure 6(b)). Thus, ERMES might be involved in the distribution of hexadecenal along mitochondria and condition the localization of the Hfd1p enzyme. Furthermore, we confirmed that hexadecenal toxicity is enhanced in ERMES mutants (Figure 6(c)), which suggests that a normal Hfd1p distribution at mitochondria might be necessary to efficiently counteract hexadecenal-induced growth arrest. Of note, ER-mitochondria contacts are also marks for mitochondrial fission mediated by Dnm1p [48], and ERMES has been implied in this process [49]. Thus, a regulated transfer of hexadecenal from the ER to mitochondria at specific tethers could locally induce fragmentation of the organelle.

### 3.4. Genetic Control of the Sphingolipid Degradation Pathway via the Hog1p MAP Kinase

**Figure 7:**
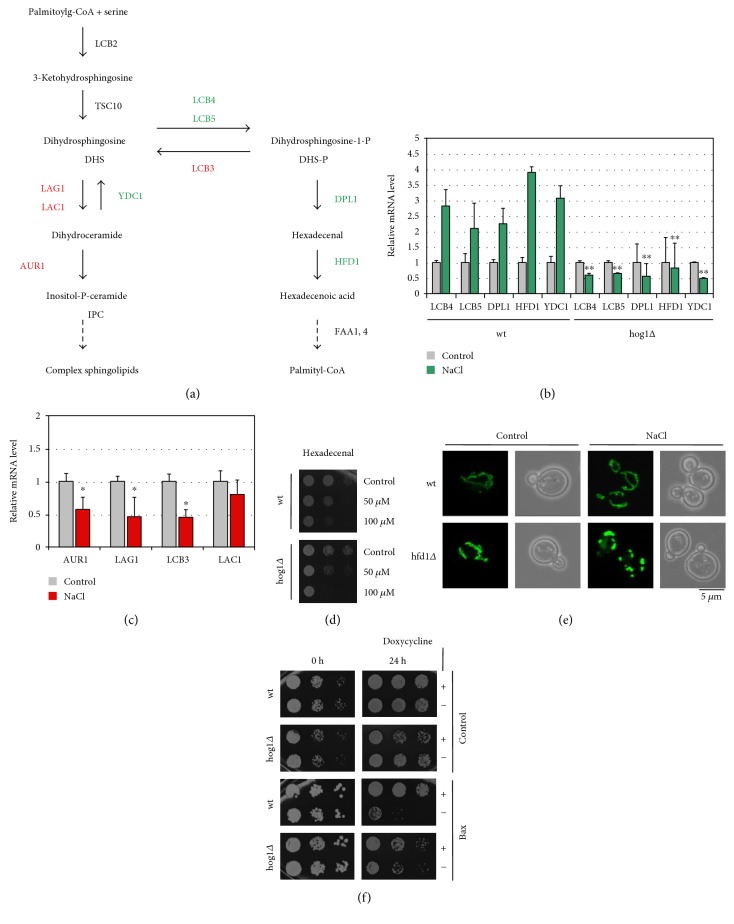
Stress regulation of the sphingolipid degradation pathway via Hog1p and its impact on mitochondrial integrity. (a) Schematic overview of the enzymatic conversions implied in sphingolipid biosynthesis and degradation in yeast. (b) Expression of sphingolipid degradation enzymes is stimulated upon salt stress in a Hog1p-dependent manner. RT-PCR analysis of gene expression in the indicated yeast strains upon salt shock (0.4 M NaCl, 20 min). Relative mRNA levels of the indicated genes were normalized for the *ACT1* control. (c) Expression of sphingolipid biosynthesis enzymes is generally repressed upon salt stress. Yeast wild-type cells were analyzed by RT-PCR as in (b). In (b, c), data are presented as mean ± SD. Three biological replicates were analyzed. Significant changes with respect to the wild type (b) or to the nonstress condition (c) are marked. ^∗^*P* < 0.05; ^∗∗^*P* < 0.01 (Student's *t*-test). (d) Loss of Hog1p function causes hexadecenal sensitivity. Growth inhibition of the indicated yeast strains by hexadecenal was assessed as in Figure 3(a). (e) Salt stress induces mitochondrial fragmentation in *hfd1Δ* mutant cells. Yeast cells expressing mt-GFP on synthetic glucose medium were treated or not with 1 M NaCl before visualization of mitochondria. (f) Loss of Hog1p function counteracts Bax inhibition. Human Bax expression was induced for 24 h as in Figure 5(b) in wild type and *hog1Δ* mutants. Growth was then recorded on YPD agar plates.

The expression of the *HFD1* gene is activated by salt and oxidative stress. Thus, we tested whether the expression of genes encoding the enzymes of sphingolipid degradation was generally regulated by stress (Figure 7(a)). We found that all enzymatic steps from ceramide to hexadecenoic acid were inducible by NaCl stress at the mRNA level (Figure 7(b)). Moreover, the Hog1p stress-activated kinase, which is the key regulator of the adaptive response of yeast to osmotic stress [50], was indispensable for this transcriptional activation. In turn, most of the reverse enzymatic reactions from S1-P to ceramide were transcriptionally repressed under the same conditions (Figure 7(c)). Thus, yeast cells employ a genetic regulation, which seems to favor the degradation of sphingolipids over its biosynthesis upon stress. This switch is controlled by the Hog1p MAP kinase, which could therefore be a modulator of sphingolipid metabolic intermediates during exposure to salt stress. Accordingly, we found that *hog1Δ* mutants were more sensitive to external hexadecenal (Figure 7(d)), which could be explained by a decreased detoxification capacity of this strain-lacking Hfd1p activation.

We further investigated the correlation between salt stress, sphingolipid metabolism, and mitochondrial function. As salt stress induced the genes encoding sphingolipid-metabolizing enzymes, a block after hexadecenal formation could be deleterious especially upon NaCl stress. We found indeed that *hfd1Δ* mutant cells showed complete mitochondrial fragmentation upon salt shock, which was not observed in the wild type (Figure 7(e)). Finally, we tested whether Hog1p function was important for Bax toxicity. We found that *hog1Δ* mutants were more resistant to Bax expression than wild-type cells (Figure 7(f)). Taken together, the Hog1p stress-activated kinase is involved in the induction of sphingolipid metabolic enzymes and this activation could lead to the accumulation of bioactive intermediates, especially hexadecenal, at mitochondria. In the absence of detoxification by the Hfd1p enzyme, this can induce the mitochondrial death pathway by favoring the activity of proapoptotic proteins such as Bax. Of note, hyperosmotic and NaCl stress are known environmental conditions, which induce mitochondria-mediated cell death in yeast [51, 52]. In this scenario, stress-activated Hog1p could trigger mitochondrial dysfunction by activating sphingolipid degradation. This model is in agreement with the previous finding that sustained activation of Hog1p impairs mitochondrial respiration, increases ROS production, and induces cell death [53].

It is important to note that the sphingolipid metabolite hexadecenal also inhibits mitochondrial function in higher eukaryotes. Hexadecenal addition to murine mitochondria efficiently permeabilizes the outer membrane [17], which has been postulated as an early irreversible decision to enter the mitochondria-mediated death pathway [19]. Accordingly, hexadecenal is a potent activator of apoptosis in mouse and human cell lines [18].

## 4. Conclusions

Our work suggests that the modulation of mitochondrial activity and induction of cell death by hexadecenal are a conserved feature of eukaryotic cells. However, apart from its proapoptotic function when externally applied to cells or mitochondria, it was not known whether intracellular regulation of hexadecenal metabolism was involved in modulating mitochondrial function and death. Importantly, we show here that the regulation of the sphingolipid degradation pathway is a decisive trigger of mitochondrial function and cell growth in yeast. Hexadecenal is the intermediate in this pathway with the highest impact on mitochondrial function. Genetic interventions, which favor intracellular hexadecenal production, induce ROS imbalance, decrease respiration, and arrest cell growth. Moreover, stress-activated signaling pathways, such as HOG, are implied in the regulation of sphingolipid degradation, which in turn can be decisive for the entry into cell death pathways upon environmental stress. Mutations in the ALDH3A2 gene, encoding the human ortholog of yeast Hfd1p fatty aldehyde dehydrogenase, cause Sjögren-Larsson syndrome, a rare neurocutaneous disorder [54]. In the light of our results, the implication of the accumulation of toxic fatty aldehydes and their effect on mitochondrial function should be further investigated in human cells with impaired ALDH3A2 enzymatic activity.

## Figures and Tables

**Table 1:** Yeast strains used in this study.

Name	Relevant genotype	Source
BY4741	*MATa*; *his3Δ1*; *leu2Δ0*; *met15Δ0*; *ura3Δ0*	EUROSCARF
BY4741 *hfd1*	BY4741 with *hfd1Δ::KanMX*	EUROSCARF
BY4741 *faa1*	BY4741 with *faa1Δ::KanMX*	EUROSCARF
BY4741 *faa4*	BY4741 with *faa4Δ::KanMX*	EUROSCARF
BY4741 *dpl1*	BY4741 with *dpl1Δ::KanMX*	EUROSCARF
BY4741 *fzo1*	BY4741 with *fzo1Δ::KanMX*	EUROSCARF
BY4741 *dnm1*	BY4741 with *dnm1Δ::KanMX*	EUROSCARF
BY4741 *fis1*	BY4741 with *fis1Δ::KanMX*	EUROSCARF
BY4741 *hog1*	BY4741 with *hog1Δ::KanMX*	EUROSCARF
BY4741 *om14*	BY4741 with *om14Δ::KanMX*	EUROSCARF
BY4741 *mcr1*	BY4741 with *mcr1Δ::KanMX*	EUROSCARF
BY4741 *rdl1*	BY4741 with *rdl1Δ::KanMX*	EUROSCARF
BY4741 *mdm10*	BY4741 with *mdm10Δ::KanMX*	EUROSCARF
BY4741 *mdm12*	BY4741 with *mdm12Δ::KanMX*	EUROSCARF
BY4741 *ybh3*	BY4741 with *ybh3Δ::KanMX*	EUROSCARF
BY4741 *msn2msn4*	BY4741 with *msn2Δ::Nat msn4Δ::KanMX*	E. de Nadal
BY4741 Hfd1-GFP Om14-dsRed	BY4741 with plasmids pAG426-GPD-HFD1-GFP (*URA3*) + pAG415-GPD-OM14-dsRed	This study
BY4741 GAL1-ccdB-HA	BY4741 with plasmid pAG426-GAL1p-ccdB-HA (*URA3*)	This study
BY4741 GAL1-DPL1-HA	BY4741 with plasmid pAG426-GAL1p-DPL1-HA (*URA3*)	This study
*hfd1* GAL1-ccdB-HA	BY4741 *hfd1Δ::KanMX* with plasmid pAG426-GAL1p-ccdB-HA (*URA3*)	This study
*hfd1* GAL1-DPL1-HA	BY4741 *hfd1Δ::KanMX* with plasmid pAG426-GAL1p-DPL1-HA (*URA3*)	This study
W303-1A	*MATa*; *ade2-1*; *his3-11,-15*; *leu2,3-112*; *ura3-1*; *trp1*	R. Rothstein
W303-1A Bax	W303-1A with plasmid pCM189-Bax (Tet_Off_-Bax-c-myc, *URA3*)	This study
W303-1A Bcl-x_L_	W303-1A with plasmid pCM184-Bcl-x_L_ (Tet_Off_-Bcl-x_L_-c-myc, *TRP1*)	This study
W303-1A pCM189	W303-1A with plasmid pCM189	This study
W303-1A pCM184	W303-1A with plasmid pCM184	This study
BY4741 Bax	BY4741 with plasmid pCM189-Bax (Tet_Off_-Bax-c-myc, *URA3*)	This study
*hfd1* Bax	BY4741 *hfd1Δ::KanMX* with plasmid pCM189-Bax (Tet_Off_-Bax-c-myc, *URA3*)	This study
*hog1* Bax	BY4741 *hog1Δ::KanMX* with plasmid pCM189-Bax (Tet_Off_-Bax-c-myc, *URA3*)	This study
BY4741 pCM189	BY4741 with plasmid pCM189	This study
*hfd1* pCM189	BY4741 *hfd1Δ::KanMX* with plasmid pCM189	This study
*hog1* pCM189	BY4741 *hog1Δ::KanMX* with plasmid pCM189	This study
BY4741 Ybh3-GFP	BY4741 with plasmid pUG36-GFP-YBH3 (*URA3*)	This study
BY4741 Hfd1-GFP Mmm1-mCherry	BY4741 with plasmids pAG426-GPD-HFD1-GFP (*URA3*) + pAG413-GAL1-MMM1-mCherry (*HIS3*)	This study
*mdm10* Hfd1-GFP	BY4741 *mdm10Δ::KanMX* with plasmid pAG426-GPD-HFD1-GFP (*URA3*)	This study
*mdm12* Hfd1-GFP	BY4741 *mdm12Δ::KanMX* with plasmid pAG426-GPD-HFD1-GFP (*URA3*)	This study
*mmm1* Hfd1-GFP	BY4741 *mmm1Δ::KanMX* with plasmid pAG426-GPD-HFD1-GFP (*URA3*)	This study
BY4741 mt-GFP	BY4741 with plasmid pVT100U-mtGFP (*URA3*)	B. Westermann
*dnm1* mt-GFP	BY4741 *dnm1Δ::KanMX* with plasmid pVT100U-mtGFP (*URA3*)	This study
*fis1* mt-GFP	BY4741 *fis1Δ::KanMX* with plasmid pVT100U-mtGFP (*URA3*)	This study
